# *Neoboletusantillanus* sp. nov. (Boletaceae), first report of a red-pored bolete from the Dominican Republic and insights on the genus *Neoboletus*

**DOI:** 10.3897/mycokeys.49.33185

**Published:** 2019-03-29

**Authors:** Matteo Gelardi, Claudio Angelini, Federica Costanzo, Francesco Dovana, Beatriz Ortiz-Santana, Alfredo Vizzini

**Affiliations:** 1 Via Angelo Custode 4A, I-00061 Anguillara Sabazia, RM, Italy; 2 Via Cappuccini 78/8, I-33170 Pordenone, Italy; 3 National Botanical Garden of Santo Domingo, Santo Domingo, Dominican Republic; 4 Department of Life Sciences and Systems Biology, University of Turin, Viale P.A. Mattioli 25, I-10125 Torino, Italy; 5 US Forest Service, Northern Research Station, Center for Forest Mycology Research, One Gifford Pinchot Drive, Madison, Wisconsin 53726, USA

**Keywords:** Boletales, molecular phylogeny, Greater Antilles, neotropical boletes, *
Sutorius
*, taxonomy

## Abstract

*Neoboletusantillanus***sp. nov.** appears to be the only red-pored bolete known from the Dominican Republic to date. It is reported as a novel species to science based on collections gathered in a neotropical lowland mixed broadleaved woodland. A detailed morphological description, color images of fresh basidiomes in habitat and line drawings of the main anatomical features are provided and relationships with phylogenetically and phenotypically similar taxa are discussed. Three genomic regions (nrITS, nrLSU/28S and *rpb2*) have been sequenced in order to reinforce the recognition of the new species and to elucidate its taxonomic affiliation within *Neoboletus*.

## Introduction

In recent times the intensive use of molecular tools applied to the investigation of the systematics of boletoid mushrooms and related groups (order Boletales) has dramatically revolutionized traditional classifications based on morphological traits, facilitating the research process and leading to the establishment of a novel scientific approach with unexpected taxonomic implications (Bruns and Palmer 1989, Binder 1999, Binder and Bresinsky 2002, Binder et al. 2005, Binder and Hibbett 2006, Nuhn et al. 2013, Wu et al. 2014).

In particular, members of the Boletaceae have undergone an extensive reassessment and several new genera have arisen from large, unwieldy and definitely polyphyletic assemblages such as *Boletus* Fr., *Xerocomus* Quél. and *Tylopilus* P. Karst, just to name a few (Wu et al. 2016b). Among these genera, *Neoboletus* Gelardi, Simonini & Vizzini has recently been segregated from *Boletus* s.l. (Vizzini 2014), to include taxa orbiting around the generic type *Boletusluridiformis* Rostk. that were traditionally assigned to either the polyphyletic Boletussect.Luridi Fr. emend. Lannoy & Estadès (Lannoy and Estadès 2001), Boletussect.Erythropodes Galli pro parte (Galli 2007) or Boletussubg.Luridellussect.Immutabiles and sect. Luridiformes pro parte (nom. inval., art. 39.1) (Watling and Hills 2005). Species included in *Neoboletus* are characterized by boletoid to rarely secotioid habit, tomentose to velvety pileus, yellow-olive tubes, brownish, red to orange or more rarely yellow pores, stipe surface usually finely dotted-punctate, yellowish context, tissues quickly and intensely bluing on handling or exposure, mild taste, olive-brown spore print, ellipsoid-fusiform, smooth basidiospores, trichodermal pileipellis consisting of filamentous hyphae, hymenophoral trama of the “*Boletus*-type”, fertile caulohymenium, inamyloid hyphae in the stipe trama, gymnocarpic ontogenetic development and ectomycorrhizal (ECM) association with members of the Pinaceae and Fagaceae (Vizzini 2014, Simonini and Vizzini 2015, Bessette et al. 2016, Wu et al. 2016a). The separation of *Neoboletus* from *Boletus* s. str. and its establishment at the generic rank is phylogenetically strongly supported (Binder and Hibbett 2006, Mello et al. 2006, Halling et al. 2007, 2015; Desjardin et al. 2009, Li et al. 2011, Zeng et al. 2012, Gelardi et al. 2013, 2015; Nuhn et al. 2013, Trappe et al. 2013, Arora and Frank 2014, Vizzini et al. 2014, Wu et al. 2014, Zhao et al. 2014, 2015; Zhu et al. 2014, Chakraborty et al. 2015, Simonini and Vizzini 2015, Smith et al. 2015, Urban and Klofac 2015, Henkel et al. 2016, Liang et al. 2016, Orihara and Smith 2017), the genus being tentatively placed in the “Pulveroboletus group” (Wu et al. 2014), although its taxonomic placement within the Boletaceae still remains uncertain (Nuhn et al. 2013, Wu et al. 2014).

In contrast to the well-known bolete heritage of North America, Europe and to a lesser degree East Asia, the diversity of the fleshy pored mushrooms in the neotropical forests of Central America and adjacent regions have received only relatively limited attention (e.g. Dennis 1970, Singer et al. 1983, 1990, 1991, 1992; Gómez and Singer 1984, Singer and Gómez 1984, Halling 1989, 1997; Gómez 1997, Halling et al. 1999, 2004, 2008, 2012a, b; Flores Arzù and Simonini 2000, Franco-Molano et al. 2000, Halling and Mueller 2002, 2003, 2005; Mata et al. 2003, Mueller et al. 2006, Halling and Ortiz-Santana 2009, Flores Arzù et al. 2012, García-Jiménez 2013, although there are many others). Particularly the Caribbean appear to be little explored from the mycological perspective; information is generally widely dispersed and members of the Boletales (including also lamellate and sequestrate representatives) have only sporadically been reported over the past two centuries (Berkeley and Curtis 1869, Hitchcock 1898, [Bibr B91][Bibr B92]; Murrill 1910, 1918, 1921; Baker and Dale 1951, Dennis 1970, Kreisel 1971, Singer and Fiard 1976, Reid 1977, Hosford and Trappe 1980, Alphonse 1981, Pegler 1983, 1987; Miller et al. 2000, Guzmán et al. 2004, Camino Vilaró et al. 2006, Ortiz-Santana 2006, Courtecuisse and Welti 2013, Lécuru and Courtecuisse 2013, Moreau et al. 2013). In the Dominican Republic (Hispaniola), as far as the boletoid fungi are concerned and aside from the recent settlement of the genus *Phylloporopsis* Angelini et al. based on *Phylloporusboletinoides* A.H. Smith & Thiers (Farid et al. 2018) and a few other reports of boletes annotated in general publications (Minter et al. 2001, Lodge et al. 2001), the monographic treatment of Ortiz-Santana et al. (2007) currently remains the sole and as yet most comprehensive taxonomic account dealing with the Boletaceae and Suillaceae for this country.

*Neoboletusantillanus* is described herein as a new species to science using morphological and three-loci (nrITS, nrLSU/28S and *rpb2*) molecular data, based on multiple collections from a lowland mixed woodland consisting of a number of different neotropical broadleaved trees, in purported ECM association with the widespread, natively sand-growing littoral seagrape, *Coccolobauvifera* (L.) L. (Polygonaceae), a small woody plant naturally distributed throughout the Caribbean basin (Séne et al. 2015, 2018). This notable species appears to be the first and sole red-pored bolete recorded in the Dominican Republic so far and one of the very few ECM members of the Boletaceae to be found in local lowland deciduous forested ecosystem.

The present paper is one in a series of intended contributions devoted to the study of neotropical Boletales, aiming to provide new insights into the taxonomy, phylogenetic relationships, plant and substrate associations, ecological importance, conservation and biogeographic patterns of the bolete communities occurring in the Dominican Republic, with continued biodiversity investigations of underexplored areas.

## Materials and methods

### Collection site and sampling

Specimens examined were collected in a hilly forest near the cemetery of Sousa, in Puerto Plata Province, Dominican Republic, and are deposited in the Herbarium of Jardín Botánico Nacional of Santo Domingo, Dr. Rafael Ma. Moscoso (JBSD) (acronym from Thiers 2019), while “ANGE” and ‘‘MG’’ refer to the personal herbarium of Claudio Angelini and Matteo Gelardi, respectively. Herbarium numbers are cited for all collections from which morphological features were examined. Author citations follow the Index Fungorum, Authors of Fungal Names (www.indexfungorum.org/authorsoffungalnames.htm).

### Morphological studies

Macroscopic descriptions and ecological information, such as habitat notations, time of fruiting and associated plant communities accompanied the detailed field notes of the fresh basidiomata. Color terms in capital letters (e.g. Myrtle Green, pl. VIII) are from Ridgway (1912). Photographs of collections were taken in the natural habitat using a Nikon Coolpix 8400 digital camera. Microscopic anatomical features were observed and recorded from revived dried material; sections were rehydrated either in water, 5% potassium hydroxide (KOH) or in anionic solution saturated with Congo red. All anatomical structures were observed and measured from preparations in anionic Congo red. Colors and pigments were described after examination in water and 5% KOH. Measurements were made at 1000× using a calibrated ocular micrometer (Nikon Eclipse E200 optical light microscope). Basidiospores were measured directly from the hymenophore of mature basidiomes, dimensions are given as (minimum) average ± standard deviation (maximum), Q = length/width ratio with the extreme values in parentheses, Qm = average quotient (length/width ratio) ± standard deviation and average spore volume was approximated as a rotation ellipsoid [V = (π.L.W2)/6 ± standard deviation]. The notation [n/m/p] indicates that measurements were made on “n” randomly selected basidiospores from “m” basidiomes of “p” collections. The width of each basidium was measured at the widest part, and the length was measured from the apex (sterigmata excluded) to the basal septum. Metachromatic, cyanophilic and iodine reactions were tested by staining the basidiospores in Brilliant Cresyl blue, Cotton blue and Melzer’s reagent, respectively. Line drawings of microstructures were traced in free-hand based on digital photomicrographs of rehydrated material.

### DNA extraction, PCR amplification and DNA sequencing

Genomic DNA was isolated from 10 mg of four dried herbarium specimen (Table 1), by using the DNeasy PlantMini Kit (Qiagen, Milan Italy) according to the manufacturer’s instructions. PCR amplifications were performed with the primers ITS1F/ITS4 for the nrITS region (White et al. 1990, Gardes and Bruns 1993), LR0R and LR5 for the nrLSU region (Vilgalys and Hester 1990) and the reverse complement of bRPB2-6R2 (Matheny et al. 2007) and bRPB2-7.1R2 (5'– CCCATNGCYTGYTTVCCCATDGC –3') or RPB2-B-F1 and RPB2-B-R (Wu et al. 2014) for partial *rpb2*. Amplification reactions were performed in a PE9700thermal cycler (Perkin-Elmer, Applied Biosystems) following Vizzini et al. (2015). The PCR products were purified with the AMPure XP kit (Beckman Coulter) and sequenced by MACROGEN Inc. (Seoul, Republic of Korea). The sequences were submitted to GenBank (http://www.ncbi.nlm.nih.gov/genbank/) and their accession numbers are reported in Table 1.

**Table 1.** Samples sequenced for the present study.

Species	GenBank acc. number			Source, date and country
	nrITS	nrLSU (28S)	*rpb2*	
* Neoboletus antillanus *	MK388290	MK388302	MK488082	JBSD127417 (holotype), 14/12/2014, Dominican Republic
* Neoboletus antillanus *	MK388291	MK388302	–	JBSD127416, 03/12/2013, Dominican Republic
* Neoboletus antillanus *	MK388292	–	–	JBSD127418, 01/12/2017, Dominican Republic
* Boletus brunneopanoides *	MK388293	MK512677	–	BOS 389 (CFMR, holotype), 21/10/2002, Belize

### Sequence alignment, data set assembly and phylogenetic analyses

**Figure 1.**
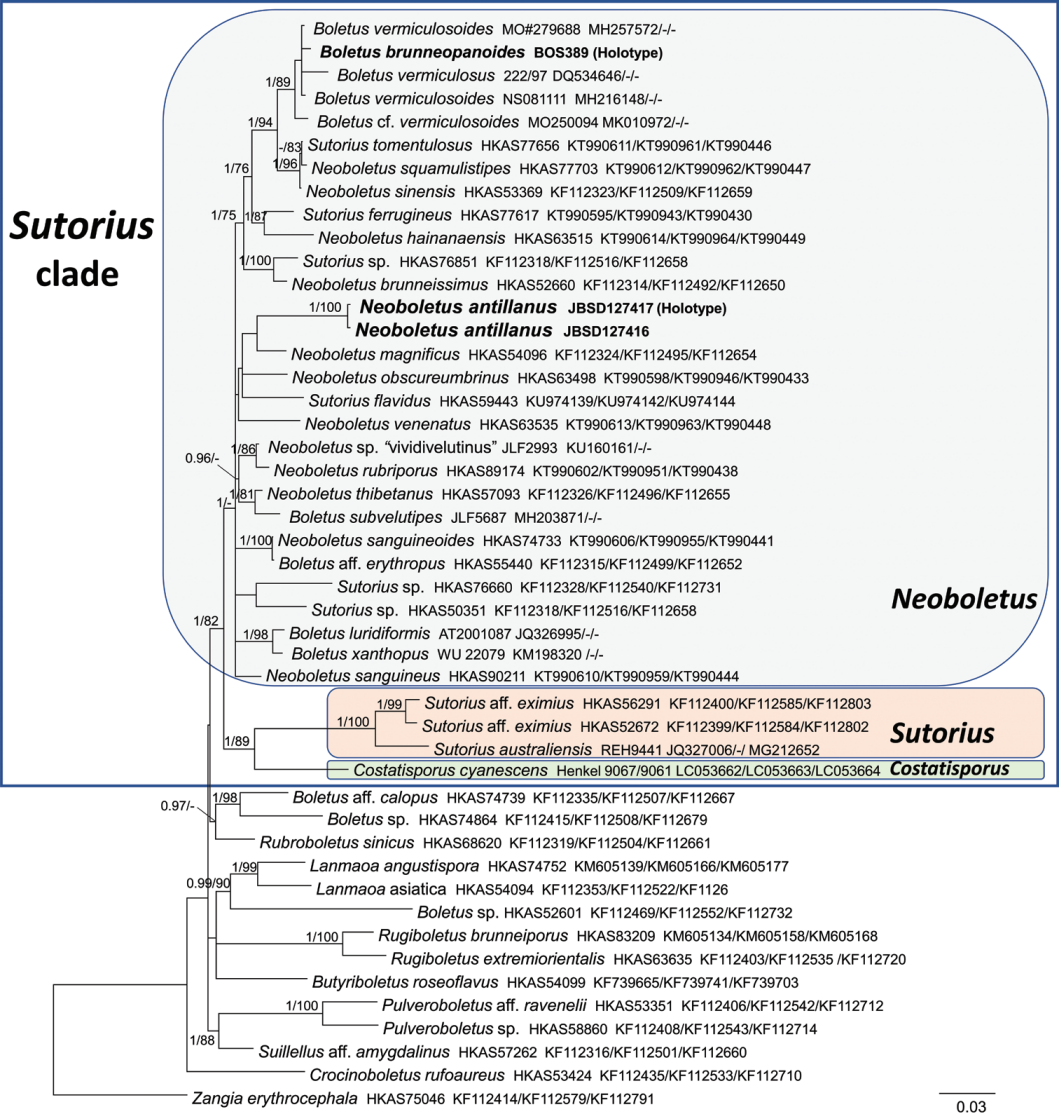
Phylogeny of the *Pulveroboletus* group based on a Bayesian and Maximum-likelihood inference analysis of a matrix of concatenated sequences from three nuclear gene regions (nrLSU/28S, *rpb1* and *rpb2*). *Zangiaerythrocephala* was used as outgroup taxon. Values for clades that are supported in either the Bayesian (posterior probabilities, BPP) and Maximum likelihood (ML bootstrap percentage, MLB) analyses are indicated. BPP ≥ 0.95 and MLB ≥ 70% are given above clade branches. Newly sequenced collections are boldfaced in black. For each collection, the specific epithet (as present in GenBank), the herbarium code and GenBank accession numbers of the nrLSU/*rpb1*/*rpb2* sequences are reported.

The sequences obtained in this study were checked and assembled using Geneious v. R 11.1.4 (Kearse et al. 2012) and compared to those available in GenBank database (https://www.ncbi.nlm.nih.gov/genbank/) by using the BLASTN algorithm (Altschul et al. 1990). A general combined Maximum likelihood tree including all the Boletaceae sequences present in GenBank and UNITE (http://unite.ut.ee/) databases was generated to detect the phylogenetic position of our collections in the major clades of Boletaceae as circumscribed by Wu et al. (2014) (tree not shown). Consequently, phylogenetic analyses were restricted to the major clade including *Neoboletus* sequences (*Pulveroboletus* group, Fig. 1).

Our datasets consist of sequences of *Neoboletus* and other sequences with greatest similarity available in GenBank selected based on BLASTN search and previous molecular studies including *Neoboletus* collections (Wu et al. 2014, 2016a, b; Smith et al. 2015; Urban and Klofac 2015).

Sequences were aligned with MAFFT v. 7.017 (Katoh et al. 2002) and then manually adjusted using Geneious v. R 11.1.4 (Kearse et al. 2012). Two phylogenetic analyses were performed: the first phylogenetic analysis, based on a combined nrLSU/*rpb1*/*rpb2* dataset, was focused on the intergeneric position of the new species in the *Pulveroboletus* group of the Boletaceae, as delimited by Wu et al. (2014). According to the results by Wu et al. (2014), *Zangiaerythrocephala* was chosen as outgroup taxon for the three-loci combined dataset. The second phylogenetic analysis based only on a nrITS sequence dataset was restricted to the taxa closely related to the new species (genus *Neoboletus*). *Costatisporuscyanescens* was used as outgroup taxon for this dataset following Smith et al. (2015).

**Figure 2.**
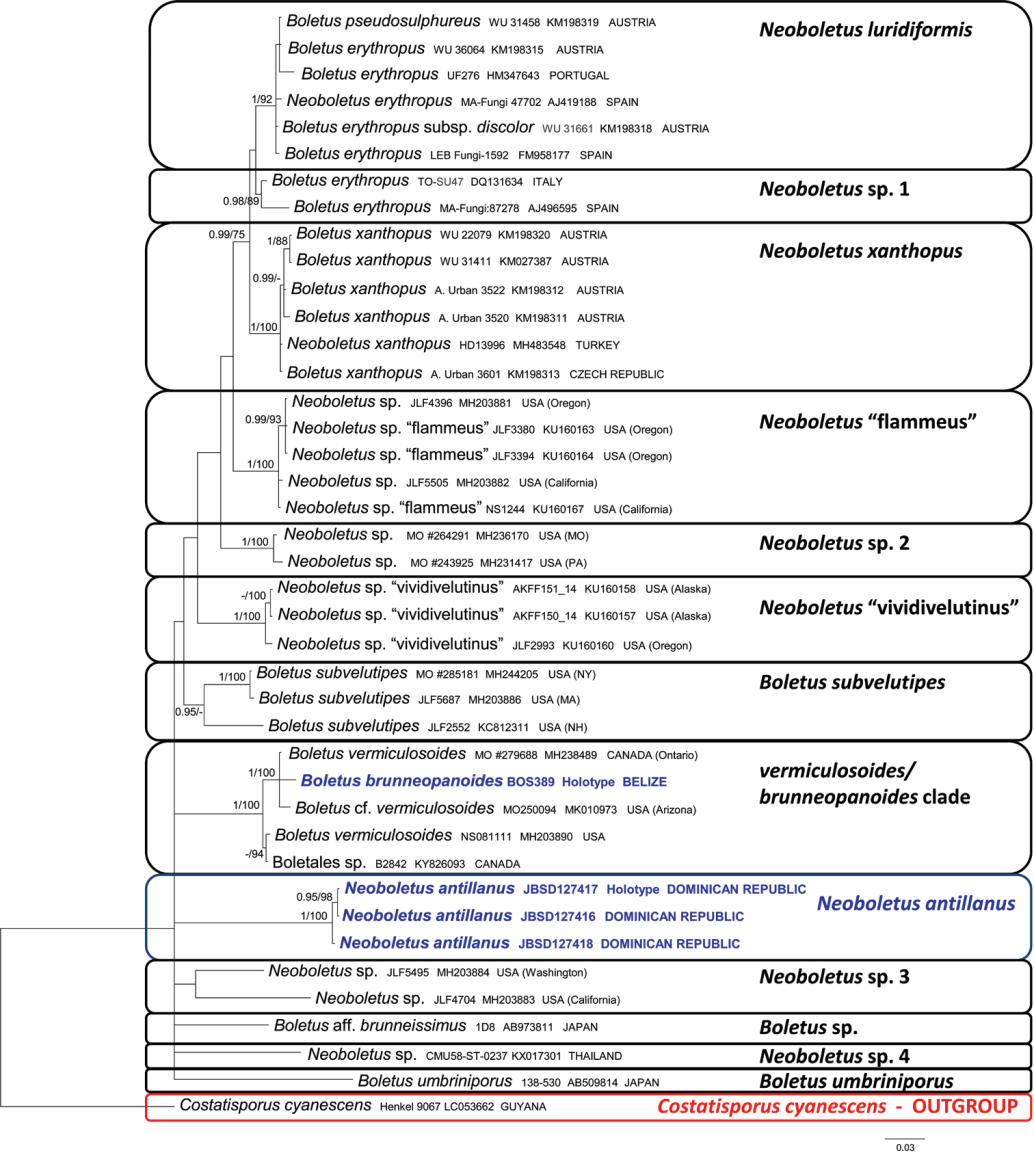
Bayesian phylogram obtained from the nrITS sequence alignment of *Neoboletus* species. *Costatisporuscyanescens* was used as outgroup taxon. Values for clades that are supported in either the Bayesian (posterior probabilities, BPP) and Maximum likelihood (ML bootstrap percentage, MLB) analyses are indicated. BPP ≥ 0.95 and MLB ≥ 70% are given above clade branches. Newly sequenced collections are boldfaced in blue. For each collection, the specific epithet (as present in GenBank), the herbarium code, GenBank accession number of the nrITS sequence and geographical origin (country) are reported.

The GTRGAMMA model of sequence evolution was selected for both analyses. The two phylogenetic analyses were inferred with three partitions: nrLSU(28S)/*rpb1*/*rpb2* and ITS1/5.8S/ITS2, respectively. The datasets were analyzed using Bayesian inference (BI) and Maximum likelihood (ML) criteria. The BI was performed with MrBayes v.3.2 (Ronquist et al. 2012) with four incrementally heated simultaneous Monte Carlo Markov Chains (MCMC) run for 10 million generations, under the selected evolutionary model. Trees were sampled every 1000 generations, resulting in overall sampling of 10001 trees; the first 2500 trees were discarded as “burn-in” (25 %). For the remaining trees, a majority rule consensus tree showing all compatible partitions was computed to obtain estimates for Bayesian Posterior Probabilities (BPP). The ML was performed with RAxML v.7.2.8. (Stamatakis 2006) and a total of 1000 bootstrap replicates (Felsenstein 1985) were computed to assess the relative robustness of the branches. Only BPP values ≥ 0.95 and MLB (Maximum likelihood bootstrap) values ≥ 70 % have been reported in the phylogenetic trees (Figs 1, 2). Pairwise % identity values of the sequences were calculated using Geneious v. R 8.1.2 (Kearse et al. 2012). Alignments and phylogenetic trees are available at Tree-BASE (www.treebase.org, submission number S24011).

## Results

### Molecular analysis

The combined nrLSU/*rpb1*/*rpb2* data matrix (focused on the *Pulveroboletus* group) comprised 47 sequences and is 2381 bp long. The nrITS data matrix (focused on *Neoboletus*) comprised 41 sequences and is 830 bp long. As the topology and branches support values of all the analyses are consistent, only the Bayesian trees with both BPP and MLB values are shown (Figs 1, 2). In the combined analysis (Fig. 1) a major clade is recognizable (BPP = 1, MLB = 82%), here named as the *Sutorius* clade, where the two sister (BPP = 1, MLB = 89%) genera *Sutorius* and *Costatisporus* are sister (BPP = 1, MLB = 82%) to the genus *Neoboletus*. The two collections of the new species clustered together within the genus *Neoboletus* forming a strongly supported clade (BPP = 1, MLB = 100%) which is sister (with no support) to *N.magnificus*. In the nrITS analysis (Fig. 2) the three collections of the new species (*N.antillanus*) clustered together in a strongly supported clade (BPP = 1, MLB = 100%) which shows no clear phylogenetic affinities with other species. The three nrITS sequences (677 to 683 bp) and the two nrLSU sequences (840 to 857 bp) of *N.antillanus* show a pairwise % identity value of 99.7 and 100, respectively. The type specimen of *Boletusbrunneopanoides* from Belize forms: i) a strongly supported clade (BPP = 1, MLB = 89%) with also two collections of *B.vermiculosoides*, one collection of Boletuscf.vermiculosoides and one of *B.vermiculosus*, in the combined analysis; ii) a strongly supported clade (BPP = 1, MLB = 100%) with also two collections of *B.vermiculosoides*, one collection of Boletuscf.vermiculosoides and one of Boletales sp. (KY826093), in the nrITS analysis.

### Taxonomy

#### Neoboletus antillanus

Taxon classificationFungiBoletalesBoletaceae

Angelini, Gelardi, Costanzo & Vizzini sp. nov.

MB829549

Figs 3 , 4

##### Etymology.

the specific epithet *antillanus* (Latin) refers to the occurrence of the species in the Antilles islands of the Caribbean.

##### Original diagnosis.

Basidiomes stipitate-pileate with tubular hymenophore characterized by medium-small size, pinkish red to reddish pileus surface, orange-red pores, reddish orange to purple-red punctuations on a yellow stipe surface, golden yellow strigosity at the stipe base, yellow context, tissues bruising dark blue when injured or exposed, ellipsoid-fusiform, smooth basidiospores, ixocutis pileipellis consisting of gelatinized, repent filamentous hyphae and occurrence in neotropical lowland mixed broadleaved forests in putative ECM association with host species (*Coccolobauvifera*) other than Fagaceae and Pinaceae.

##### Holotypus.

DOMINICAN REPUBLIC, Municipality of Sousa, Puerto Plata Province, Loc. Cemetery, 19°44'40"N, 70°32'21"W, 100 m a.s.l., 14 Dec 2014, C. Angelini (JBSD127417; *isotypus* ANGE434 and MG719).

**Figure 3.**
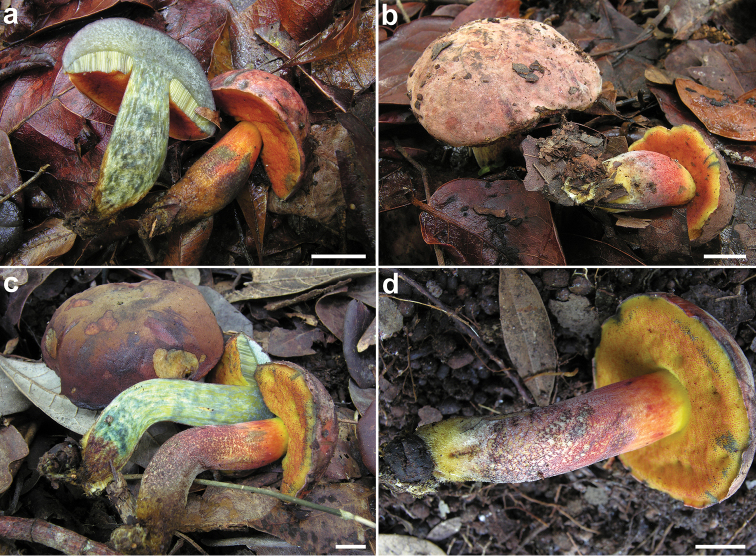
*Neoboletusantillanus*. Basidiomata in situ. **a** JBSD127416 **b** JBSD127417 (holotype) **c, d** JBSD127418. Scale bars: 1 cm. Photos by C. Angelini.

*Basidiomes* medium-small (Fig. 3). *Ontogenetic development* gymnocarpic. *Pileus* (3.0) 3.5–7.5 (8.0) cm broad, at first hemispherical then persistenly convex and finally broadly pulvinate-flattened, sometimes slightly depressed at centre, regularly to hardly unevenly shaped, moderately fleshy, firm at the beginning but progressively softer with age, flabby in old basidiomes; margin steady to faintly wavy-lobed, initially involute then curved downwards, extending beyond the tubes up to 1 mm; surface matt, dry but slightly greasy with moist weather, very finely tomentose, not cracked; cuticle somewhat variable in color, ranging from wine red, dark red or reddish pink to pastel pink (Pomegranate Purple, pl. XII; Spinel Red, pl. XXVI; Pinkish Vinaceous, pl. XXVII; Carmine, Eosine Pink, Geranium Pink, Rose Doree, pl. I; Alizarine Pink, Jasper Pink, Old Rose, pl. XIII), gradually fading with age and becoming pinkish cream to pale ochraceous pink (Flesh Pink, pl. XIII; Pale Ochraceous-Salmon, Pale Ochraceous-Buff, Light Buff, Light Ochraceous-Buff, Warm Buff, pl. XV) with olive-brown to brownish shades (Dresden Brown, pl. XV; Olive Lake, pl. XVI; Light Yellowish Olive, Buffy Olive, pl. XXX) tending to progressively spread from the center towards the peripheral zone; slowly bluing (Methy Green, Sea Green, Prussian Green, pl. XIX; Motmot Blue, Capri Blue, pl. XX) on handling or when injured; subcuticular layer cream-yellowish (Citrine Yellow, pl. XVI). *Tubes* at first thin then increasingly broader and as long as or slightly longer than the thickness of the pileus context at maturity (up to 1.0 cm long), adnate but soon deeply depressed around the stipe apex, occasionally subdecurrent, bright yellow (Lemon Chrome, pl. IV) to olive-yellow (Yellowish Citrine, pl. XVI), turning blue (Prussian Green, Duck Green, Invisible Green, pl. XIX) when cut and eventually fading to drab yellowish (Aniline Yellow, Pyrite Yellow, pl. IV). *Pores* initially forming a concave then flat surface, at first small then gradually wider (up to 1 mm in diam.), simple, roundish to barely angular at maturity, at first bright orange-red to orange (Scarlet Red, Scarlet, pl. I) although concolorous with the tubes (Lemon Chrome, pl. IV) towards the margin, soon becoming yellowish orange (Flame Scarlet, Orange Chrome, pl. II) and finally yellowish olive (Yellowish Citrine, pl. XVI) with very pale orange hues (Orange, pl. III), quickly and intensely turning blue (Prussian Green, Duck Green, Invisible Green, pl. XIX) on bruising or when injured. *Stipe* (3.5) 4.0–9.0 (9.5) × (1.0) 1.5–2.0 (2.5) cm, longer than or as long as the pileus diameter at maturity, central to slightly off-center, solid, firm, dry, straight or curved, at first ventricose-fusiform, later cylindrical but either sligthly swollen towards the base to decidedly clavate or tapering downwards, not to barely rooting, evelate; surface at the apex or in the upper third usually smooth to occasionally very faintly reticulate due to the sub-decurrence of the hymenophore in some specimens and bright yellow (Lemon Chrome, pl. IV) to lemon yellow (Strontian Yellow, pl. XVI), elsewhere showing a fine, purple-red, dark red to orange-red (Indian Lake, pl. XXVI; Amaranth Purple, pl. XII; Carmine, Scarlet Red, pl. I) punctuation (Fig. 2d) partly hiding the bright yellow (Lemon Chrome, pl. IV) ground color; the base is typically wrapped by a conspicuous golden yellow to brownish yellow strigosity (Fig. 2d) (Raw Sienna, pl. III; Yellow Ocher, pl. XV); bruising greenish blue (Light Blue Green, Blue Green, Forest Green, pl. XVII) throughout when pressed; *basal mycelium* golden yellow (Raw Sienna, pl. III; Yellow Ocher, pl. XV). *Context* firm when young, later soft textured and eventually flabby in the pileus (up to 1.0 cm thick in the central zone), a little more fibrous in the stipe, lemon yellow (Strontian Yellow, pl. XVI) throughout, usually with purple-brown (Indian Lake, pl. XXVI; Amaranth Purple, pl. XII) spots in the stipe, especially at the extreme base; turning blue (Methy Green, Sea Green, Prussian Green, pl. XIX; Motmot Blue, Capri Blue, pl. XX) more or less evenly when exposed to air and finally fading to drab yellowish (Aniline Yellow, Pyrite Yellow, pl. IV); subhymenophoral layer lemon yellow (Strontian Yellow, pl. XVI). *Odour* and *taste* not distinctive. *Spore-print* not obtained but likely olive-brown.

**Figure 4.**
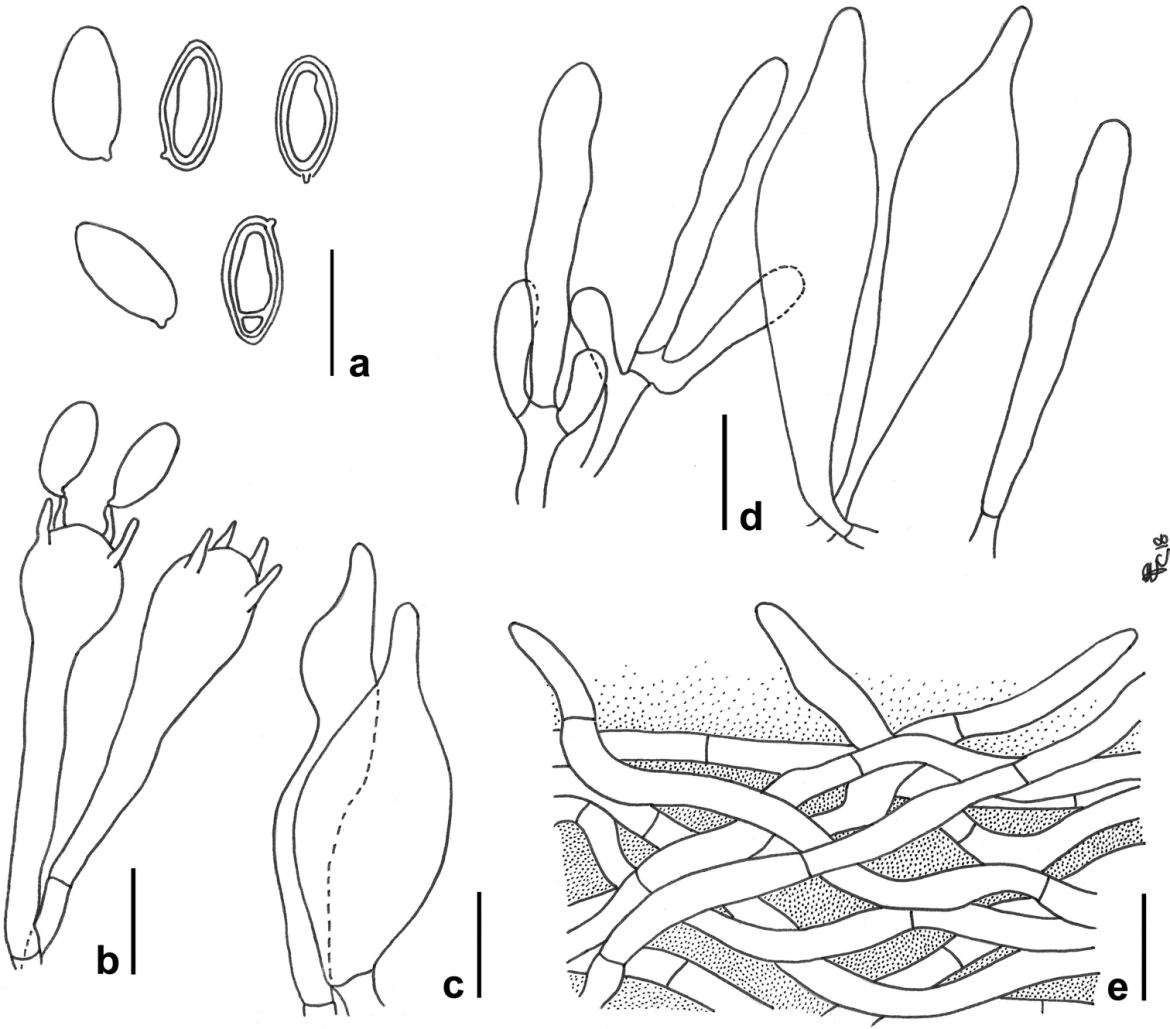
*Neoboletusantillanus*. Micromorphological features (JBSD127417) **a** Basidiospores **b** Basidia **c** Caulocystidia **d** Cheilo- and pleurocystidia **e** Elements of the pileipellis. Scale bars: 10 μm (**a–d**); 20 μm (**e**). Drawings by F. Costanzo.

*Basidiospores* [102/5/3] (8.8) 11.1 ± 0.78 (12.7) × (4.1) 4.9 ± 0.26 (6) μm, Q= (1.85) 1.96–2.54 (2.57), Qm= 2.24 ± 0.12, V= 143 ± 23 μm³, inequilateral, ellipsoid-fusiform to ellipsoid in side view, ellipsoid in face view, smooth, apex rounded, with a short apiculus and with a shallow suprahilar depression, moderately thick-walled (0.5–0.9 μm), honey yellow colored in water and 5% KOH, having one or two large oil droplets when mature, rarely pluri-guttulate, inamyloid to very faintly dextrinoid, acyanophilic and with an ortochromatic to very faint metachromatic reaction (Fig. 4a). *Basidia* 24–48 × 10–13 μm (n= 26), cylindrical-clavate to clavate, moderately thick-walled (0.5–0.8 μm), predominantly 4-spored but also 1- or 2-spored, usually bearing relatively short sterigmata (2–6 μm), hyaline to pale yellowish and containing straw-yellow oil guttules in water and 5% KOH, bright yellow (inamyloid) in Melzer’s, without basal clamps (Fig. 4b); basidioles subcylindrical to faintly clavate, similar in size to basidia. *Cheilocystidia* (19) 21–56 × 4–9 (11) μm (n= 23), very common, moderately slender, projecting straight to sometimes flexuous, irregularly cylindrical or cylindrical-fusiform, fusiform to narrowly lageniform, showing a narrow and long neck, with rounded to subacute tip, smooth, moderately thin- to slightly thick-walled (0.3–0.9 μm), hyaline to pale yellowish in water and 5% KOH, bright yellow (inamyloid) in Melzer’s, without epiparietal encrustations (Fig. 4d). *Pleurocystidia* (41) 44–55 × 5–11 μm (n= 14), uncommon, shape, color and chemical reactions similar to but usually longer than cheilocystidia (Fig. 4d). *Pseudocystidia* not recorded. *Pileipellis* (Fig. 4e) an ixocutis consisting of strongly interwoven, elongated, filamentous and sinuous, frequently branched, repent to occasionally erect hyphae heavily embedded in gelatinous matter; terminal elements 20–72 × 3–9 μm, long and slender, cylindrical, apex pointed, moderately thick-walled (up to 1 μm), pale yellow to golden yellow in water and 5% KOH, inamyloid in Melzer’s, smooth to sometimes ornamented by a subtle zebra-like epiparietal encrustation; subterminal elements similar in shape, size and color to terminal elements. *Stipitipellis* a texture of slender, parallel to subparallel and longitudinally running, smooth-walled, adpressed hyphae, 3–11 μm wide, hyaline to yellowish in water and 5% KOH; the stipe apex covered by a well-developed caulohymenial layer consisting of sterile clavate caulobasidioles, abundant, predominantly 4- or 2-spored, fertile caulobasidia and projecting, irregularly cylindrical or cylindrical-fusiform, ventricose-fusiform to fusiform, sublageniform to rarely short mucronate *caulocystidia* (Fig. 4c) similar in shape and color to but slightly broader than hymenial cystidia, (23) 25–45 (54) × 5–13 (15) μm (n= 16), having a wall up to 0.8 μm thick. *Lateral stipe stratum* under the caulohymenium present and well differentiated from the stipe trama, of the “boletoid type”, at the stipe apex a (20) 30–40 (50) μm thick layer consisting of divergent, inclined and running towards the external surface, loosely intermingled and branched hyphae remaining separate and embedded in a gelatinous substance. *Stipe-trama* composed of densely arranged, subparallel to moderately interwoven, frequently septate, cylindrical to filamentous, smooth, inamyloid hyphae, 4–13 μm broad. *Basal tomentum hairs* 40–150 μm thick, consisting of tightly adpressed, parallel to subparallel, septate, filamentous, occasionally branched, relatively thick-walled (up to 0.8 μm) hyphae, 2–5.5 μm wide, terminal elements with blunt apex, pale yellow to honey yellow in water and 5 % KOH. *Hymenophoral trama* bilateral divergent of the “*Boletus*-type”, with slightly to strongly divergent, recurved-arcuate and loosely arranged, often branched, restricted at septa, gelatinous hyphae (lateral strata hyphae in transversal section not touching each other, (2) 4–8 (10) μm apart, 3–13 μm broad), hyaline to very pale yellowish in water and 5% KOH, inamyloid in Melzer’s; lateral strata (20) 30–50 (60) μm thick, mediostratum (20) 30–60 (70) µm thick, axially arranged, consisting of a tightly adpressed, non-gelatinous bundle of hyphae, 3–10 µm broad; in Congo Red the mediostratum is darker than the lateral strata. *Thromboplerous hyphae* (= oleiferous hyphae sensu Clémençon 2004) very common and particularly frequent in the hymenophore, golden yellow in 5% KOH. *Clamp-connections* absent everywhere. *Hyphal system* monomitic.

##### Ecology.

solitary to gregarious, growing on limestone among litter in a seasonally dry and moist anthropised lowland mixed stand under a large array of neotropical broadleaved trees, including *Coccolobauvifera*, which represents its probable ECM host tree. See Parra et al. (2018) for further details on vegetation.

##### Edibility.

Unknown.

##### Examined material.

DOMINICAN REPUBLIC, Municipality of Sousa, in Puerto Plata Province, Loc. Cemetery, 19°44'40"N, 70°32'21"W, 100 m a.s.l., a single middle-aged specimen, 03 Dec 2014, C. Angelini (JBSD127416, ANGE425 and MG718); same loc., two young to mature specimens, 14 Dec 2014, C. Angelini (JBSD127417, Holotype, ANGE434 and MG719, Duplo); same loc., several dozens of specimens, most of which heavily parasitized by *Hypomyces* sp., 01 Dec 2017, C. Angelini (JBSD127418, ANGE958 and MG720).

##### Known distribution.

Presently only known from the type locality in the Dominican Republic (Greater Antilles, Caribbean).

## Discussion

### *Neoboletusantillanus* phylogeny and interspecific relationships

Phylogenetic analyses corroborate the proposal of the new species *N.antillanus* (Figs 1, 2). It forms an independent evolutive line within *Neoboletus* with no evident phylogenetic relationships (it is sister to the Chinese *N.magnificus* in the combined analysis, but without statistical support) to allied congeneric taxa. According to the same analyses, *B.brunneopanoides*, a Belizean red-pored bolete species phylogenetically nested in *Neoboletus*, clustered in the same clade with collections named *B.vermiculosus*/*B.vermiculosoides* from North America. Should future molecular work prove conspecificity among these three species, *B.vermiculosus* Peck would have priority.

### Taxonomic circumscription of *N.antillanus*

The genus *Neoboletus* currently encompasses fewer than ten species geographically restricted to the northern hemisphere and essentially distributed in temperate and tropical regions. However, judging from morphological traits, there might be an additional number of species, up to three times as many in fact, belonging to the same genus, most of which have not yet been molecularly investigated. It is worth noting that a group of Chinese researchers after having firstly accepted *Neoboletus* as an independent genus (Wu et al. 2016a), have subsequently reduced it in synonymy with *Sutorius* Halling, Nuhn & Fechner based on a wider interpretation of the generic boundaries within the Boletaceae (Wu et al. 2016b). As previously pointed out by Gelardi (2018), we presently disagree with this broad circumscription of *Sutorius* since it is, judging from the original description, easily separated from *Neoboletus* based on the overall dark colors, different stipe ornamentation pattern, different spore print color, pores stuffed in early developmental stages like those of *Boletus* s. str. and *Butyriboletus* Arora & J.L. Frank and non-bluing tissues (Halling et al. 2012b). Enough, in our opinion, to state they are not the same thing especially because they cluster in two different (although with a low statistical support) sister clades. Moreover, molecular studies carried out by Smith et al. (2015) on false-truffle fungi from north-eastern South America (Guyana) and our nrLSU/*rpb1*/*rpb2* analysis (Fig. 1) indicate the sequestrate genus *Costatisporus* T.W. Henkel & M.E. Smith as the sister taxon to *Sutorius*. *Costatisporus*, *Neoboletus* and *Sutorius* form the *Sutorius* clade (Fig. 1).

*Neoboletusantillanus* is easily identifiable among other species of the same genus based on the following set of unique morphologically informative features: 1) medium-small size, 2) reddish to pinkish red then pinkish cream pileus surface, 3) pores orange red to yellowish orange, 4) stipe ornamented over the lower three fourth by purple-red to reddish orange punctuations on a yellow background, 5) lowermost part of the stipe prominently strigose with golden yellow to brownish yellow hairs, 6) yellow context, 7) tissues bruising dark blue when injured, 8) ellipsoid-fusiform, smooth basidiospores, 9) ixocutis pileipellis consisting of gelatinized, repent filamentous hyphae and 10) occurrence in neotropical lowland mixed broadleaved forests. To date, *N.antillanus* has never been found with host species other than local autoctonous broadleaved trees and does not appear to be associated with either Pinaceae or Fagaceae (the latter plant family is not present in Dominican Republic). Moreover, such a purported ECM association of *N.antillanus* with the endemic *C.uvifera* might implicate a neotropical origin. Further suggestion supporting a symbiotic relationship between *N.antillanus* and *C.uvifera* is the co-occurrence at the same locality with *Cantharelluscoccolobae* Buyck, P.-A. Moreau and Courtec., which is strictly associated with seagrape in tropical America (Buyck et al. 2016).

Among the other endemic red-pored boletes reported from Central America, *Boletuspyrrhosceles* Halling, *B.guatemalensis* R. Flores & Simonini, *B.dupainii* Boudier and *B.paulae* J. García, Singer & F. Garza-Ocañas superficially resembles *N.antillanus*. However, *B.pyrrhosceles* is easily separated by the reddish brown to brownish orange pileus surface, adnate to subdecurrent hymenophore, shallow tubes (up to 5 mm deep), brownish red pores, tomentose and reticulate stipe that is entirely brownish red to deep red, slightly smaller basidiospores (9.1–11.2 × 4.2–4.9 μm, Qm= 2.3), trichodermal pileipellis and association with *Quercushumboldtii* Bompl. in Colombia (Halling 1992). *Boletusguatemalensis* has a whitish to pale yellow context with yellowish green spots towards the stipe base, radially elongated angular pores, stipe surface with brownish green fibrils in the lower half and a smooth, whitish base, white basal mycelium, unchanging tissues, mostly 2- or 3-spored basidia, a cutis pileipellis consisting of non-gelatinized hyphae, hymenophoral trama intermediate between the “*Boletus*-type” and the “*Phylloporus*-type” and is associated with *Pinuscaribaea* Morelet in Guatemala, Belize and Mexico (Flores Arzù and Simonini 2000, Ortiz-Santana et al. 2007, García-Jiménez et al. 2013). *Boletusdupainii* s. Ortiz-Santana et al. differs in the larger size (pileus up to 13 cm broad), polish and shiny, carmine red to crimson red pileus surface, deep red pores, stipe base devoid of strigosity, longer spores (12.8–14.4 × 4–5.6 μm, Qm= 2.9), smaller basidia (24–29.6 × 9.6–10.4 μm), shorter pleuro-, cheilo- and caulocystidia (26.4–47.2 × 7.2–8.8 μm, 16–30.4 × 4.8–8 μm and 16–36.8 × 5.6–11.2 μm, respectively), thinner pileipellis hyphae (up to 6.5 μm diam.) and growth in symbiosis with *Quercus* spp. in Belize (Ortiz-Santana et al. 2007). This species has recently been assigned to *Rubroboletus* Kuan Zhao & Zhu L. Yang on account of morphological and molecular evidence (Zhao et al. 2014). It was originally described from Europe (Boudier 1902) where it appears to be widespread although uncommon, but in recent times it has also been reported from the New World (McConnell and Both 2002, Ortiz-Santana et al. 2007, Both et al. 2009, García-Jiménez 2013, Bessette et al. 2016). However, the conspecificity of the European material with that from the western hemisphere is yet to be confirmed and a comparative analysis is currently under examination. Finally, *B.paulae* exhibits a deep red, vinaceous red to strawberry red pileus surface, smooth stipe base, whitish gray basal mycelium, pale whitish yellow and erratically bluing context on exposure, hymeniform pileipellis consisting of chains of inflated to subglobose elements up to 34 μm broad and ECM association with oaks in Mexico (García-Jiménez et al. 2013).

Although *N.antillanus* exhibits some superficial morphological affinities with *Boletusvermiculosus* Peck, *B.vermiculosoides* A.H. Smith & Thiers and *B.brunneopanoides* B. Ortiz, these three species have larger basidiome size (pileus 7–18 cm broad and stipe 9–14 cm long in *B.vermiculosus*, pileus up to 12 cm and 16 cm broad in *B.vermiculosoides* and *B.brunneopanoides*, respectively), subtomentose to velvety, yellowish brown or grayish brown to dark brown pileus surface, brownish orange to amber brown or dark brown pore surface fading brownish yellow with age, extremely fine brownish punctuations on stipe surface and stipe base without hairs. *B.vermiculosus* also differs from *N.antillanus* in the trichodermal pileipellis devoid of gelatinous matter, longer basidiospores [(11) 12.6–14 (15) × (4) 4.9–5.6 (6) μm, Qm= 2.6] and the occurrence under Fagaceae. *B.vermiculosoides* is further distinguished by the paler, whitish-yellow stipe surface, narrower basidiospores [9–12 × 3–3.5 (4) μm], smaller basidia (20–26 × 7–9 μm) and association with Fagaceae, whereas *B.brunneopanoides* is also separated by the whitish stipe surface, narrower basidiospores (8.8–12.8 × 4 μm), smaller basidia (20.4–32× 8–8.8 μm) and the occurrence with Pinaceae (*P.caribaea*) (Coker and Beers 1943, Smith and Thiers 1971, Both 1993, Bessette et al. 2000, 2016; Halling and Mueller 2005, Ortiz-Santana et al. 2007). *Boletusvermiculosus* and *B.vermiculosoides* were originally described from eastern North America but the former is also encountered in Central America south to Belize and Costa Rica (Bessette et al. 2000, 2016; Halling and Mueller 2005), while *B.brunneopanoides* was only found in Belize (Ortiz-Santana et al. 2007). Up to now, neither of these species has been reported from the Dominican Republic.

At least two additional North American boletes might be confused with *N.antillanus*, namely *Boletussubluridus* (Murrill) Murrill and *B.fairchildianus* (Singer) Singer. The combination of yellowish orange, orange-pink to purplish red pileus surface, dark red pores, non-strigose stipe base, slightly longer basidiospores [(8.5) 9–14(14.5) × (3.5) 4–6(7) μm], smaller basidia (20–25.5 × 7.5–10 μm and occurrence with oaks and pines in south-eastern USA differentiate *B.subluridus* from *N.antillanus* (Murrill 1938, Singer 1945, 1947, both as B.miniato-olivaceusvar.subluridus Singer; Both 1993, Bessette et al. 2000; 2016), whereas *B.fairchildianus* is distinguished by the larger size (pileus up to 15 cm broad), stipe base without strigosity, larger basidiospores [(12.5) 13–18.8 (19.7) × (4.5) 5–8 μm] and the association with *Quercus* spp. in south-eastern USA and Mexico (Singer 1945, 1947, both as B.rubricitrinusvar.fairchildianus Singer; Both 1993, Bessette et al. 2000, 2016; García-Jiménez 2013).

*Neoboletusluridiformis* (Rostk.) Gelardi, Simonini & Vizzini (= *Boletuserythropus* Pers. s. Fr. et auct. p.p. non s. Pers.) differs significantly from *N.antillanus* in the large sized basidiomes (pileus up to 25–30 cm in diam.), dark chocolate brown to umber brown, velvety pileus surface, bright red pores, stout, fleshy stipe (up to 15 × 8 cm), non-strigose stipe base, longer basidiospores [(12.8) 13.3–15.5 (16.5) × 4.2–5.5 μm, Qm= 2.95], trichodermal pileipellis with interwoven erect, non-gelatinous hyphae and occurrence in Europe in temperate regions (Pilát and Dermek 1974, Alessio 1985, Breitenbach and Kranzlin 1991, Lannoy and Estadès 2001, Muñoz 2005, Watling and Hills 2005, Klofac 2007, Knudsen and Taylor 2012; pers. obs.).

The eastern Asian species *N.brunneissimus* (W.F. Chiu) Gelardi, Simonini & Vizzini and *N.antillanus* share some common features such as basidiome size, presence of golden yellow to brownish yellow strigosity at the stipe base, yellowish context and dark blue staining of tissues by auto-oxidation but the former is readily separated by the velvety and rusty brown to umber-brown pileus cuticle, rusty brown to reddish-brown pores, denser and rusty-brown punctuation on stipe surface, trichoderm pileipellis consisting of non-gelatinized erect hyphae with slightly shorter and narrower terminal elements [23–45 (58) × 3.5–5 (7) μm] and the occurrence in East Asia in association with Fagaceae and Pinaceae (Chiu 1948, 1957; Bi et al. 1997, Mao 2000, 2009; Wang and Liu 2002, Wang 2004, Wang et al. 2004; Zang 2006, Wu et al. 2016a, Gelardi 2018).

The Chinese *N.magnificus* (W.F. Chiu) Gelardi, Simonini & Vizzini, *Sutoriussanguineoides* G. Wu & Zhu L. Yang and *S.sanguineus* G. Wu & Zhu L. Yang are three additional eastern Asian species that may be confused with *N.antillanus*. Aside from the different geographical distribution and the ECM deciduous and coniferous host associates (Fagaceae and Pinaceae), the former species is also delimited by the dark red to reddish brown pores in the early developmental stages, a decidedly clavate to bulbous stipe base (up to 6 cm broad) that is devoid of or sometimes with inconspicuous strigosity and non-gelatinized trichodermal pileipellis with broader end elements (up to 16 μm wide) (Chiu 1948, 1957; Bi et al. 1997, Mao 2000, 2009; Wang 2004, Wang et al. 2004, Zang 2006, Wu et al. 2016a), whereas *S.sanguineoides* and *S.sanguineus* are both separated from *N.antillanus* on account of the deep red, blood red to brownish red pileus surface, dark red to brownish red pores, non-strigose stipe base, non-gelatinized trichodermal pileipellis and the occurrence in subalpine forests at very high elevations (over 3000 m alt.) (Wu et al. 2016b). Furthermore, *S.sanguineoides* differs in its decidedly larger basidiospores [13.5–17 (21) × 5–7 μm, Qm= 2.56] while *S.sanguineus* also exhibits an evenly red stipe surface, slightly longer basidiospores [10–14 (15) × 5–6 (7) μm, Qm= 2.14] and broader cystidioid pileipellis terminal cells (9–15 μm wide) (Wu et al. 2016b).

## Supplementary Material

XML Treatment for Neoboletus antillanus
